# Effect of Spraying Polyurea on the Anti-Blast Performance of the Ultra-High Performance Concrete Slab

**DOI:** 10.3390/s22249888

**Published:** 2022-12-15

**Authors:** Bin Gao, Jun Wu, Qinyi Chen, Jun Yu, Haitao Yu

**Affiliations:** 1 School of Urban Rail Transportation, Shanghai University of Engineering Science, Shanghai 201620, China; 2 School of Civil Engineering, Southeast University, Nanjing 211189, China; 3 Department of Geotechnical Engineering, Tongji University, Shanghai 200092, China

**Keywords:** polyurea, UHPC slabs, strengthening effectiveness, near-field explosion, parametric analysis

## Abstract

Recently, polyurea has been applied to improve the anti-blast performance of metal plates, masonry walls, and concrete structures. However, the strengthening effectiveness of polyurea on ultra-high performance concrete (UHPC) slabs with an overall response is still unclear. Hence, this paper examined the strengthening effectiveness of polyurea on the anti-blast performance of UHPC slabs under near-field explosion by the finite element (FE) method. First, a benchmark finite element model for UHPC and polyurea-UHPC (PUHPC) slabs under blast loading was established and validated by field blast tests, with scaled distances ranging from 0.4 m/kg^1/3^ to 0.8 m/kg^1/3^. After that, parametric analysis was conducted to fully understand the strengthening effectiveness of polyurea on the anti-blast performance of the UHPC slab. Factors including the scaled distance, polyurea thickness, span-to-depth ratio of the slab, and longitudinal reinforcement ratio were considered. The results showed that (1) spraying polyurea on the rear face of the UHPC slab can reduce the width of cracks and mitigate the damage of specimens; (2) the strengthening effectiveness of polyurea on the UHPC slab became prominent when the UHPC slab suffered a larger maximum deflection; (3) in terms of the deflection and energy absorption capacity of PUHPC slabs, the optimum thickness of sprayed polyurea was determined to be 8 mm to 12 mm; and (4) by adopting the multiple nonlinear regression method, a prediction formula was developed to quickly obtain the end rotation of the UHPC slab strengthened with polyurea under near-field explosions.

## 1. Introduction

Normal reinforced concrete (NRC) slabs are widely used as structural members in civil engineering. During their service life, NRC slabs might withstand severe dynamic loads arising from terrorist attacks, gas explosions, and industrial explosions. Under such circumstances, RC slabs are prone to suffering severe damage, which might cause the degradation of the overall stiffness of a structure and further trigger the whole structure to collapse [1,2,3]. Ultra-high-performance concrete (UHPC) has higher mechanical properties (i.e., tensile and compressive strength) than normal concrete and has been gradually applied to important bridges or building structures [4,5]. Hence, the safety of UHPC slabs under extreme loads has attracted many concerns in the engineering field.

The anti-blast performance of UHPC slabs has been widely investigated. It was found that the crack width in UHPC slabs was smaller than that in NRC slabs under the same blast loading [6]. Furthermore, because the propagation of cracks in the UHPC slab can be well-inhibited by adding steel fibers, the anti-blast performance of the UHPC slab was better than that of the NRC slab [7]. However, as the scaled distance *Z* decreased (0.35 ≤ *Z* ≤ 0.5 m/kg^1/3^, where *Z* = *R*/*W*^1/3^, *R* is the standoff distance from the charge center to the structure surface, and *W* is the charge weight of the equivalent TNT explosive), the UHPC slab suffered severe flexural failure, in which a main flexural crack tended to develop and extend from the bottom to the top of the slab [7,8]. Therefore, the fracture of the UHPC slab might have been triggered. Based on the existing results [9], it was found that although UHPC had a high compressive and tensile strength and its fracture energy was normally approximately 40 kJ/m^2^, which was less than that of the engineering cementitious composite, and its toughness was relatively low. When subjected to extreme blast loading (such as near-field explosion or contact detonation), spalling and debris splashing can be generated from UHPC slabs, possibly causing secondary damage to the surrounding personnel or facilities [10,11]. Therefore, for UHPC structures with high safety requirements, it is important to take appropriate measures to enhance their anti-blast performance.

Currently, several retrofitting methods for existing concrete structures have been proposed and implemented. Among them, steel plates and fiber reinforced polymers (FRPs) were adopted. It is obvious that placing a steel plate at the bottom of a slab is able to improve the overall flexural stiffness and strength [12,13], but the steel plate inevitably brings excessive weight to the slab, and in addition, the steel plate is vulnerable to corrosion. Alternatively, an FRP can be used to enhance the flexural strength of structural members and prevent debris from spalling. However, when the damage degree of the structural member is intensified, the FRP could suffer fracture and peeling-off failure [6,14,15]. Polyurea might be an appropriate retrofitting material, due to its convenience of construction and the good adhesive strength between polyurea and concrete [16]. Furthermore, polyurea is a strain rate hardening material that can contribute to improving the flexural properties of structural members subjected to extreme dynamic loading [17,18,19]. Therefore, as a retrofitting material, polyurea was adopted for blast mitigation of concrete structures [20,21,22,23,24], metal plates [25,26,27,28], and masonry walls [29,30].

The anti-blast capacity of concrete structures retrofitted with polyurea has recently been reported. Shi et al. [20] investigated the failure mode, the distribution of concrete fragments, and the damage degree of NRC slabs with and without spraying polyurea under contact explosion. It was observed that fragments at the bottom of the specimen were well-retained and reduced by spraying polyurea. Wang et al. [21,22] experimentally examined the effectiveness of the polyisocyanate-oxazodone (POZD) polymer for blast mitigation of an NRC slab subjected to contact detonation. The experimental results revealed that, when the rear face of the NRC slab was sprayed with the polymer, the damage to the NRC slab shifted from local penetration to coating bulging, and at the same time, the height and distribution of bulging deformation decreased with increasing polyurea thickness. Wu et al. [23] examined the anti-blast capacity of polyurea-strengthened NRC slabs under near-field explosions (0.4 ≤ *Z* ≤ 0.8 m/kg^1/3^) via a combined numerical and experimental study. The results indicated that spraying polyurea on the rear face of the NRC slab had a better reduction effect on the residual displacement than on the maximum displacement. It was also shown that the damage of the polyurea-strengthened NRC slab was formed in a narrower area, compared to the NRC slab without sprayed polyurea. When the scaled distance further decreased (i.e., the corresponding blast loading became larger), the effectiveness of polyurea for the blast mitigation of the NRC slab was more significant. More recently, Liu et al. [24] experimentally studied the effectiveness of polyurea for the blast mitigation of geopolymer-based ultra-high-performance concrete (G-UHPC) slabs under contact detonation. According to the test results, it was found that the maximum bulge depth at the rear face mainly depended on the tensile hardening property of polyurea, the polyurea thickness, and the interfacial bonding strength between the polyurea layer and subbase. Based on the existing research, it was concluded that spraying polyurea can improve the blast resistance of concrete substrates and inhibit the damage deterioration of the specimen under close-in or contact detonation. However, the main application objects of the polyurea strengthening method were NRC slabs. To the best of the authors’ knowledge, studies focusing on the effectiveness of polyurea for blast mitigation of UHPC slabs are limited. In particular, the strengthening effectiveness and mechanism of polyurea on the overall response of a UHPC slab are still unclear. Therefore, it is important to investigate the strengthening effectiveness of polyurea on a UHPC slab applied to a structure with high safety requirements.

Due to the high cost and extensive resources needed for field blast tests, it is more productive to carry out numerical simulations to verify the test and further analyze the effect of parameters based on the verified model. For example, Kong [31] adopted the finite element method (FEM) to investigate the dynamic response of a reinforced concrete slab strengthened by an aramid fiber-reinforced plastic (AFRP) sheet under blast loading, and it was reported that factors including the AFRP layer, FRP type, strengthening mode, FRP bond strength, and TNT mass determined the retrofitting effectiveness. Wu [32] established a three-dimensional finite element model to investigate the structural response and failure mode of a reinforced concrete slab under blast loading and then proposed an empirical formula to predict the damage degree of the reinforced concrete slab. Reifarth [33] investigated the strengthening effectiveness of the FRP type on reinforced concrete under explosion (0.21 ≤ Z ≤ 0.83 m/kg^1/3^) and established a prediction model via the FEM. Liu [34] adopted the FEM to investigate the influence of the polyurea layer thickness, panel thickness, concrete strength, and reinforcement ratio on the damage degree of reinforced concrete slabs strengthened with polyurea and proposed empirical formulas of the P-I diagram to quickly evaluate the damage level of PU-NRC slabs under various blast loads. In addition to the FEM used in the above studies, the differential quadrature finite element method (DQFEM) has the characteristic of directly evaluating the integrals and derivatives included in the weak form of a problem utilizing the same sets of interpolation points [35]. Hence, the DQFEM can be employed to address the free vibrational behavior of a structure [36] and the dynamic response of a structure under blast loading [37]. Additionally, Kabir [38] proposed an accurate Bézier-based multistep method to address the nonlinear vibration and post-buckling configurations of Euler–Bernoulli composite beams reinforced with graphene nano-platelets. Attributed to the simplicity and acceptable accuracy of the FEA method, it was adopted to simulate the dynamic response of UHPC and polyurea-strengthened UHPC (PUHPC) slabs under near-field explosion in the current study.

This paper aims to examine the strengthening effectiveness of polyurea on the anti-blast capacity of UHPC slabs and draw a relevant design suggestion for the application of polyurea in strengthening UHPC slabs. To this end, a benchmark finite element (FE) model of UHPC and PUHPC slabs was established and validated with the actual measurements (i.e., the damage pattern and deflection of the specimens) from field blast tests (0.4 ≤ *Z* ≤ 0.8 m/kg^1/3^). To fully understand the effectiveness of polyurea for the blast mitigation of UHPC slabs, key factors, including the scaled distance, polyurea thickness, span-to-depth ratio of the UHPC slab, and longitudinal reinforcement ratio, were further considered. Based on the energy dissipation capacity and reduction of the mid-span deflection, the optimum polyurea thickness for the blast mitigation of UHPC slabs was suggested. A prediction formula for the assessment of the end rotation of UHPC slabs with or without a polyurea layer was then developed.

## 2. Development of the Benchmark Model

The benchmark FE model of UHPC and polyurea-strengthened UHPC (PUHPC) slabs subjected to near-field explosion was established and validated based on the configuration of the field blast test. The selected key features of the field test and benchmark model are given in the following section.

### 2.1. Field Blast Tests

#### 2.1.1. Specimens

**Figure 1:**
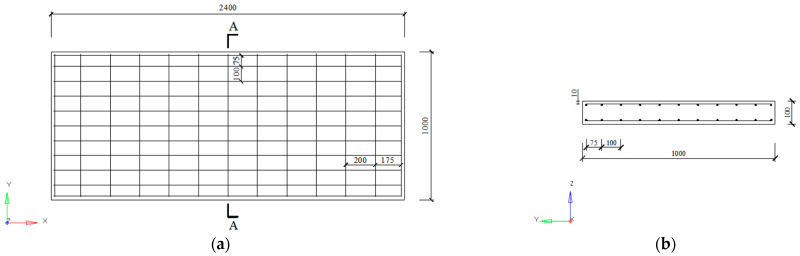
Geometric dimension and reinforcement of specimen (unit: mm): (**a**) Plan view of the UHPC slab (**b**) Cross-sectional view of the UHPC slab.

For the field blast test, five UHPC slab specimens were fabricated and tested. The detailed layout and dimensions of the specimen are reported in Figure 1, in which the slab had dimensions of 2400 mm × 1000 mm × 100 mm (length × width × thickness). Steel reinforcement with a diameter of 10 mm was fabricated in the form of an orthogonal layer and placed in the specimen. The steel reinforcement had a strength grade of HRB400, with a nominal yield strength of 400 MPa. Table 1 gives the detailed mixing proportion for the UHPC material. In previous research by our research group [39], the mechanical properties of UHPC material were tested and reported, and the corresponding experimental results are listed in Table 2. To determine the mechanical properties of the steel reinforcement, three samples were tested via a universal testing machine based on the Chinese standard GB/T 228.1-2010 [40]. Thus, Table 3 gives the mechanical properties of the steel reinforcement samples. Based on the values measured in the three tests, the average yield strength, tensile strength, elastic modulus, tangent modulus, and Poisson’s ratio of the steel reinforcement were determined to be 469 MPa, 585 MPa, 184 GPa, 864 MPa, and 0.3, respectively.

**Figure 2:**
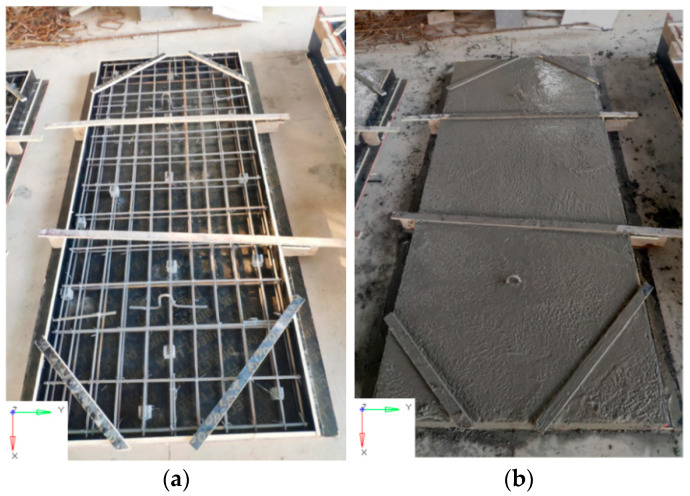
The procedure of casting UHPC slab: (**a**) Assemble steel reinforcement and fabricate formwork (**b**) Smooth the surface.

Figure 2 reports the casting of the UHPC slab. For the mixing of UHPC, a single horizontal shaft forced mixer was adopted to dry mix sand with cementitious materials (cement, silica fume, and viscosity reducing admixture) for 3 min to form a uniform mortar mixture. The water and HRWR were mixed in the container according to the mixing proportion. Then, the obtained mixture was poured slowly into the mixer and mixed for 3 min to 5 min. The steel fibers were poured into the mixer evenly and mixed for 3 min to 5 min until the steel fibers were distributed evenly. The steel fibers poured first would not be wound with the steel fibers poured later, due to the internal shear force of the mixture. In the current tests, the measured slump and diffusivity of UHPC were 230 mm and 610 mm, respectively, which meet the requirements of site construction. After completion of the fabrication, five UHPC slabs were cured at a standard temperature of 18–22 °C and relative humidity of 98.4% for 28 days.

**Figure 3:**
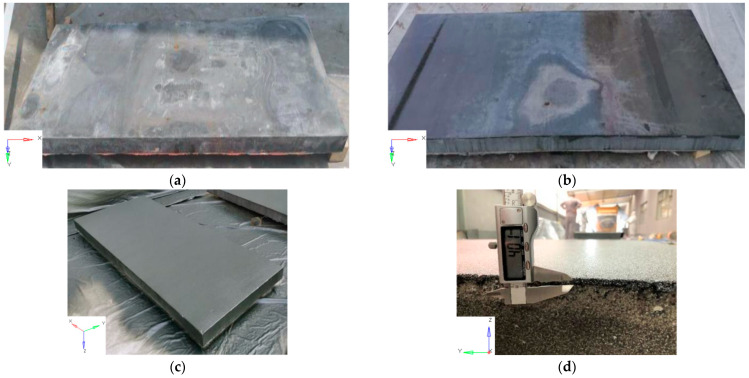
The procedure of spraying polyurea: (**a**) Polishing surface (**b**) Applying primer (**c**) Spraying polyurea (**d**) The thickness of the polyurea layer.

**Figure 4:**
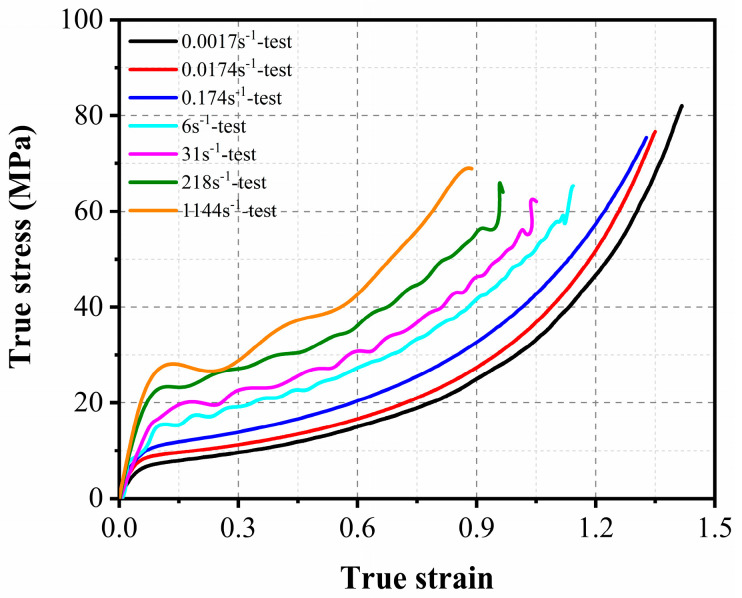
True stress-true strain curve of the polyurea.

Before spraying polyurea, three UHPC slabs were placed in a ventilated and dry environment for two weeks to remove moisture. Then, the rear face of the three UHPC slabs was strengthened by spraying polyurea with a thickness of 4 mm. The detailed spraying procedure for the UHPC slabs is described in Figure 3. According to the ASTM standard [41], the direct tensile test and split Hopkinson tension bar (SHTB) apparatus were used to obtain the dynamic tensile strength of the polyurea in the present study. Table 4 gives the mechanical properties of the polyurea. The tensile strength of the polyurea was determined to be 15 MPa under a 500 mm/min loading rate. Figure 4 illustrates the typical stress–strain response of the polyurea under tensile loading with strain rates varying from 0.0017 s^−1^ to 1144 s^−1^.

#### 2.1.2. Configuration of the Field Blast Test

**Figure 5:**
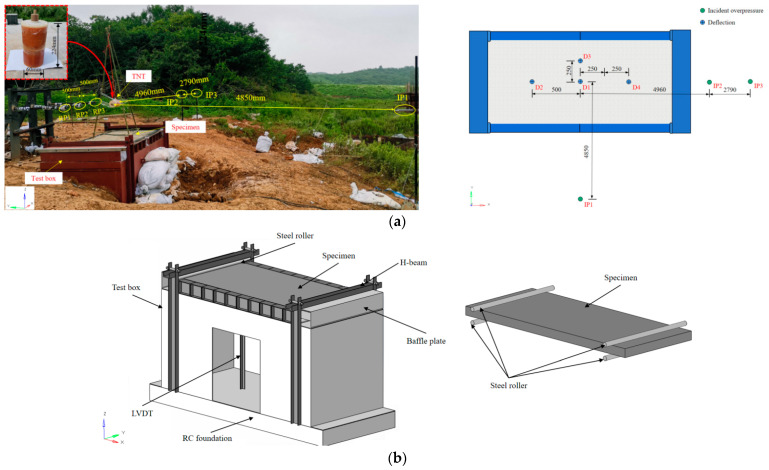
Layout of field blast test: (**a**) Setup of the test (**b**) Schematic view.

Table 5 reports the test program for the field blast test. For the name of the specimen, the notation “UHPC” represents a UHPC slab, and “PUHPC” stands for a UHPC slab strengthened with polyurea. The scaled distance *Z* can be calculated by the formula *Z* = *R*/*W*^1/3^, where *R* is the standoff distance from the charge center to the structure surface and *W* is the TNT charge weight. Figure 5 describes the arrangement of the test box and instrumentation in the field blast test. The detailed dimensions and configuration of the test box can be found in Wu [23]. In the test, the specimen had an effective length of 2000 mm. Figure 5a illustrates the setup of the instrumentation in the test. For the measurement of the blast pressure, three incident overpressure sensors (IP_1_ to IP_3_) were arranged at the same height as the center of the TNT charge. Four displacement transducers (D_1_ to D_4_) were set at the bottom of the specimen to monitor the deformation of the slab. Figure 5a also illustrates the arrangement of the TNT charge in the test, in which the explosive was placed horizontally and suspended above the center of the specimen. In each explosion, a cylindrical TNT charge with a mass of 4 kg was adopted, and at the same time, a one-end detonation was assigned to the explosion.

### 2.2. Benchmark Finite Element Model

#### 2.2.1. Numerical Model

**Figure 6:**
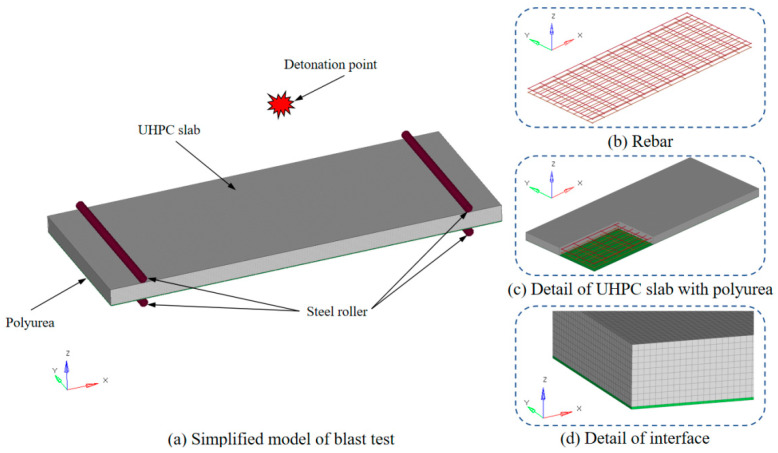
FE model of PUHPC slab under blast test.

The benchmark FE model of the UHPC and PUHPC slabs subjected to near-field explosion was established by LS-DYNA in the present study. Figure 6 describes the detailed information of the FE model of the PUHPC slab, in which the UHPC, polyurea, and supports were represented by single-point integral hexahedral solid elements, and Hughes-Liu beam elements were adopted to simulate the steel reinforcements. It was noted that the steel reinforcements were embedded into the UHPC slab by adopting the coupled algorithm of CONSTRAINED_LAGRANGE_IN_SOLID. The simple supporting condition between the specimen and the supports was simulated by the contact algorithm of SURFACE_TO_SURFACE. The full bonding behavior between UHPC and polyurea was assumed and was achieved by the common-node method in the FE model [16]. From the mesh sensitivity study [7,23], a mesh size of 10 mm was assigned to the UHPC and PUHPC slabs, and the corresponding mesh size was 10 × 10 × 2 mm for the polyurea layer in the FE model.

#### 2.2.2. Blast Loading

**Figure 7:**
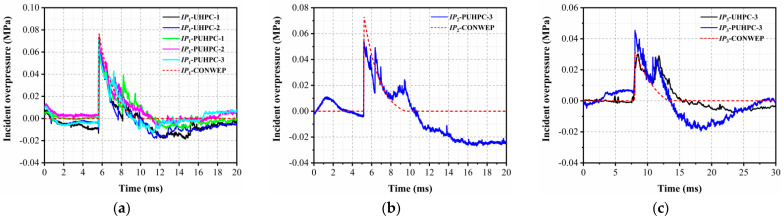
Time history of incident overpressure at collection point: (**a**) IP1 (**b**) IP2 (**c**) IP3.

The blast loading was generated by the CONWEP model in the current study. Figure 7 reports the results of the incident overpressure from the field blast test and CONWEP model, which showed that the results of the CONWEP model were close to the test results in the duration and peak overpressure at IP_1_, indicating that the incident overpressure obtained by the test was reliable. Due to the loss of the acquisition signal, the overpressure data at the IP_2_ and IP_3_ sensors for some cases were not collected. The deviation of the peak incident overpressure at IP_2_ and IP3 between the CONWEP model and field blast test was approximately 30%. This is possibly due to the fact that the shape of the TNT charge adopted in the field test was cylindrical, whereas a spherical charge was assumed in the CONWEP model [42]. However, in view of the inherent variation in the field blast test, a prediction deviation of 30% from the results of the field blast test can be considered acceptable.

#### 2.2.3. Material Models

(1) UHPC

In the current study, the dynamic behavior of the UHPC material was represented by the KCC model (Karagozian and Case Concrete model). However, the original KCC model was only applicable for simulating concrete-like materials with a cylindrical compressive strength less than 90 MPa and, thus, was inapplicable to UHPC. Therefore, when the KCC model was used to represent the UHPC material, the corresponding parameters needed to be further calibrated, including the strength surfaces, equation of state, damage function, softening indicator, and strain rate effect. The detailed formulation of the three strength surfaces of the KCC model (i.e., maximum/yield/residual strength surface) can be found in Malver [43]. In the present study, the parameters for the three strength surfaces were determined based on the triaxial compressive test of the UHPC material [44]. The parameters for the three strength surfaces of the UHPC material are given in Table 6.

The equation of state (EOS) describes the relationship between hydrostatic pressure and volumetric strain in the KCC model. The EOS parameters for the UHPC with a compressive grade of 129 MPa are summarized in Table 7. The detailed calibration of the EOS data for the UHPC material can be found in Su [7].

**Figure 8:**
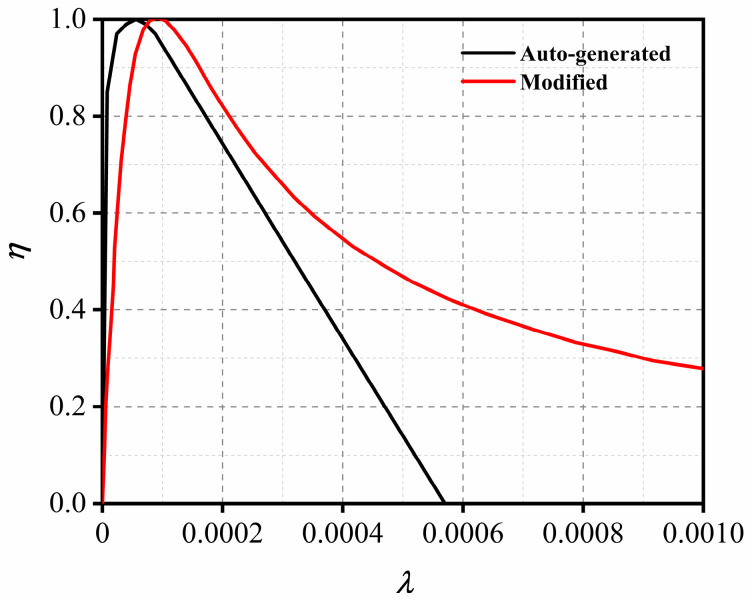
Damage Function for UHPC in current study.

The original damage function curve automatically generated by the KCC model cannot accurately describe the ductility and fracture energy of UHPC. Therefore, the damage function established by Yin [45] was adopted in this paper. Figure 8 compares the results of the damage curve between the modified and original damage functions. From the figure, it is obvious that the high ductility and fracture energy of UHPC in the post-peak stage can be well-captured by the modified damage function.

**Figure 9:**
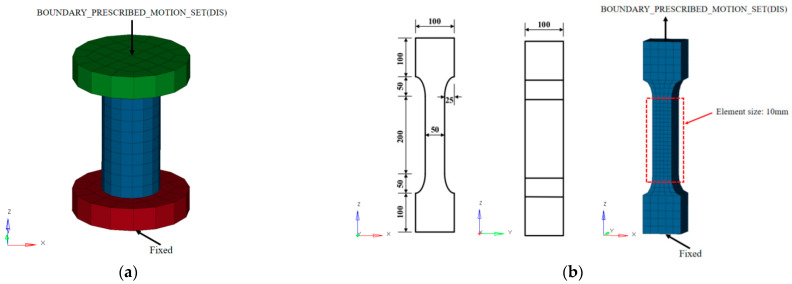
Finite element model of static test: (**a**) Uniaxial compressive test (**b**) Uniaxial tensile test (Unit: mm).

**Figure 10:**
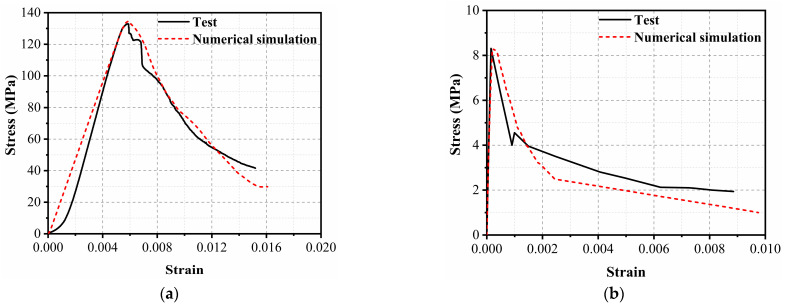
Stress–strain curves of UHPC from the test and FE simulation: (**a**) Uniaxial compression (**b**) Uniaxial tension.

The softening indicators *b*_1_ and *b*_2_ characterize the post-peak softening behavior of UHPC materials. The indicator *b*_1_ is the softening parameter under uniaxial compression and can be determined by the following process. The FE model of the uniaxial compressive test with sample dimensions of 50 mm in diameter and 100 mm in height was first established, as shown in Figure 9a. It should be noted that the sample in the FE model had the same dimension as that used in the laboratory test [46]. The FE model included the upper and lower steel plates and the UHPC sample, which were represented by a 10 mm mesh size. The uniaxial compressive test was then numerically simulated by applying a uniform downward displacement to the top node of the upper plate, while the lower plate was fully constrained. As shown in Figure 10a, when *b*_1_ equals 0.5, the FE simulation describes a stress–strain curve similar to that in the laboratory test. The determination of tensile softening indicator *b*_2_ is similar to that of *b*_1_. The FE simulation for the uniaxial tensile test of the UHPC material needed to be conducted, as shown in Figure 9b. The bottom of the specimen was completely constrained, and the simulation of the uniaxial tensile test was carried out by applying displacement on the top node of the specimen. The comparison of the stress–strain curve between the FE simulation and experimental test is reported in Figure 10b, in which *b*_2_ was determined to be −1.5 in the current study. The specific parameters in KCC model are summarized in Table A1 in Appendix A.

In the KCC model, the dynamic increase factor (*DIF*) curves of UHPC under compression and tension can be defined by Equation (1) and Equation (2), respectively, based on Ren et al. [47,48].

(1) $$DIF=\left\{\begin{array}{c} {\left(\frac{\dot{\varepsilon }}{{\dot{\varepsilon }}_{0}}\right)}^{0.014}\;\dot{\varepsilon }⩽92\\ 0.5103\times {\left(\log\dot{\varepsilon }\right)}^{2}-1.2301\times \log\dot{\varepsilon }+1.6804\;\dot{\varepsilon }>92 \end{array}\right.$$

(2) $$DIF=\left\{\begin{array}{c} {\left(\frac{\dot{\varepsilon }}{{\dot{\varepsilon }}_{0}}\right)}^{0.018}\;\dot{\varepsilon }⩽10.5\\ 11.561\times \log\dot{\varepsilon }-10.446\;\dot{\varepsilon }>10.5 \end{array}\right.$$

where $\dot{\varepsilon }$ represents the strain rate obtained from the dynamic load and $\dot{\varepsilon_{0}}$ is the strain rate obtained from the static load.

(2) Polyurea

In this study, the *MAT_SIMPLIFIED_RUBBER/FOAM model was employed to simulate the tensile behavior of polyurea under various strain rates. In this material model, the stress–strain curves of polyurea for different strain rates derived from the static and dynamic tensile tests can be input. Based on the input curves, the stress–strain response of polyurea for various strain rates can be automatically generated by the material model. The typical stress–strain curves of the polyurea used in the present study are reported in Figure 4. In addition, the density, elastic modulus, and Poisson’s ratio of polyurea were input as 1070 kg/m^3^, 120 MPa, and 0.495, respectively.

(3) Reinforcement and supports

In the current study, the *MAT_PLASTIC_KINEMATIC model was adopted to describe the mechanical properties of the steel reinforcement, in which the density, elastic modulus, and Poisson’s ratio were set to 7850 kg/m^3^, 184 GPa, and 0.3, respectively, and its corresponding yield strength was 469 MPa. It should be noted that the input material parameters of the steel reinforcement were consistent with the test results, as shown in Table 3. In addition, the input parameters of *C* and *P* were employed to consider the strain rate effects of the steel reinforcement in the FE model. For the steel reinforcement with a strength grade of HRB400 in the current study, the corresponding *C* and *P* were determined to be 40 s^−1^ and 5, respectively. The steel supports were simulated by the rigid material model, assuming that no deformation occurred for the supports.

### 2.3. Validation of the FE Model

#### 2.3.1. Damage Pattern of the Specimens

**Figure 11:**
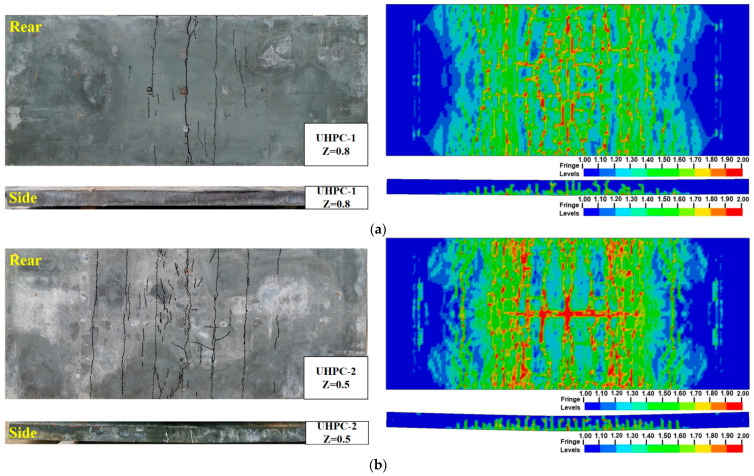

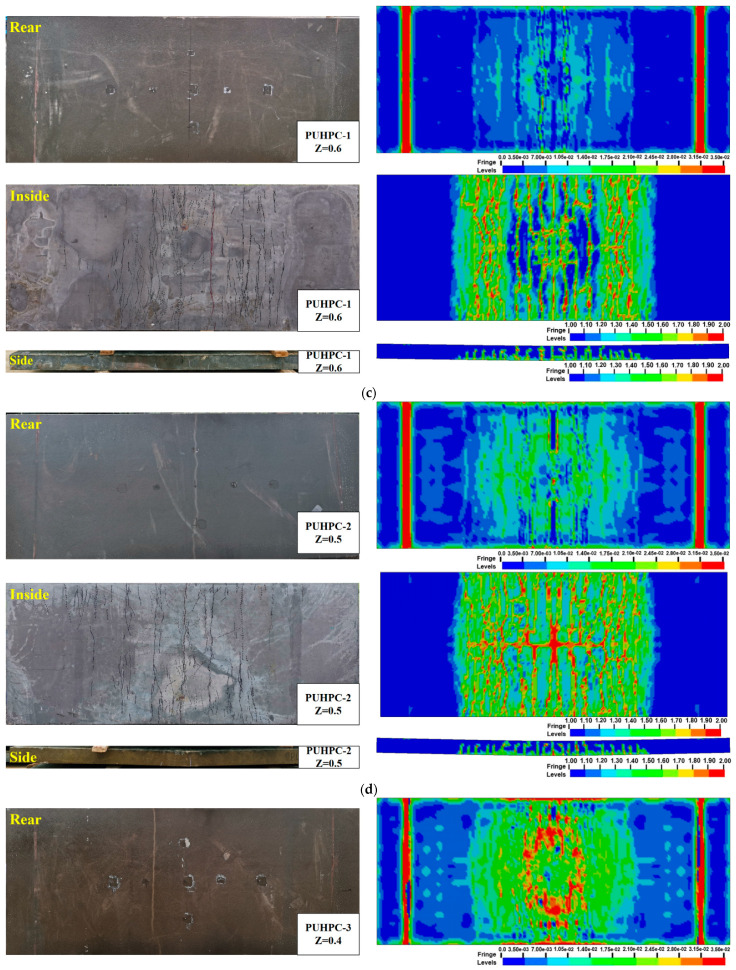

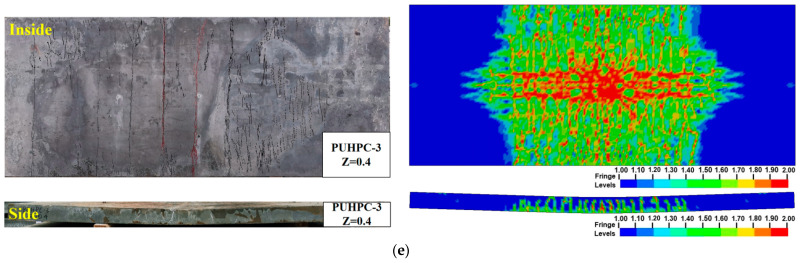
Comparison of concrete damage between test and FE method: (**a**) UHPC-1 (**b**) UHPC-2 (**c**) PUHPC-1 (**d**) PUHPC-2 (**e**) PUHPC-3.

Figure 11 reports the damage pattern of UHPC and PUHPC slabs after explosion, in which the damaged situation at the rear face and side of the specimen was given, since the cracks mainly occurred at both faces. From Figure 11a,b, the UHPC slabs mainly exhibited the flexural mode. For the UHPC-1 specimen, one major crack and few tensile cracks occurred on the rear face of the slab. For the UHPC-2 specimen, one major crack and several tensile cracks, stretching to both sides of the slab, occurred on the rear face of the slab. When the scaled distance decreased from 0.8 m/kg^1/3^ to 0.5 m/kg^1/3^, the width and distribution range of the cracks increased, which was obviously attributed to the slabs being loaded under a larger impulse. From Figure 11c–e, for the PUHPC-1, PUHPC-2, and PUHPC-3 specimens, all specimens exhibited the flexural mode. At the same time, the polyurea was stretched, but no polyurea fracture and spalling were observed at the rear face of the specimens, indicating that the polyurea had excellent bonding strength. To check the internal damage of the PUHPC slabs, the polyurea layer was peeled off after the explosion. It was found that a few major cracks, accompanied by many microcracks, developed on the rear face of the PUHPC slabs, indicating that spraying polyurea on the bottom of a UHPC slab would reduce the damage degree of the substrate and make the interior of the UHPC slab produce more microcracks to dissipate energy. When the scaled distance decreased, the distribution range of the cracks at the rear face of the specimens expanded, but the PUHPC slab still exhibited the flexural mode and more microcracks. Compared with the UHPC slab under the scaled distance of 0.5 m/kg^1/3^, the number and width of cracks on the side face of the PUHPC slab were reduced, and the width of tensile cracks on the rear face of the PUHPC slab also reduced. For the PUHPC-3 specimen subjected to the most severe explosion in the current study, the major crack at the side face did not extend to the top of the specimen, indicating that spraying polyurea can improve the blast resistance of a UHPC slab.

Figure 11 also illustrates the comparison of the damage pattern between the experimental results and numerical simulation. In the FE results, the damage of the UHPC material was represented by the damage index δ ranging from 1 to 2, representing the deterioration of the UHPC material. In presenting the damaged situation of the polyurea, the fringe level in the FE result was the effective plastic strain, and thereby, a higher mark represented a larger deformation of the polyurea. From the figure, it is shown that, under the same scaled distance, the distribution of cracks at the bottom of the PUHPC specimen was less, in comparison with that of the UHPC specimen. Figure 11c–e also show that the effective plastic strain of the polyurea was small, indicating that the tensile hardening characteristics of the polyurea might not be fully developed. Therefore, it can be postulated that the strengthening effectiveness of polyurea on UHPC may be further improved as the scaled distance decreases. It should be noted that the effective plastic strain at both sides of the polyurea was at a high level, due to the strict constraints of the rigid support. Furthermore, as shown in Figure 11c–e, both the experimental results and the FE simulation demonstrated that decreasing the scaled distance resulted in more microcracks developing at the rear face of the PUHPC slabs. The damage situation of the side face suggested that the UHPC and PUHPC slabs exhibited the flexural mode in both the experimental and FE results. Hence, according to the abovementioned analysis, it can be stated that the established FE model can reasonably predict the damage pattern of UHPC and PUHPC slabs under near-field explosion.

#### 2.3.2. Deflection of the Specimens

Table 8 summarizes the maximum and residual deflections at the mid-span of the specimens (D_1_ transducer) derived from the field blast test and FE simulation. Due to the damage of the displacement transducers, some test data were not captured; therefore, the residual deflection was manually measured for certain specimens after explosion. For PUHPC-1, the numerical deviation from the experimental residual deflection was approximately 30%. This is because the boundary condition was an ideal fixed constraint in the FE model, whereas in the field explosion test, the steel rollers at both ends of the specimen moved vertically, which caused a smaller residual deflection of the specimen. For the other specimens, the deviation of the FE result from the experimental result was less than 20% for the residual deflection.

**Figure 12:**
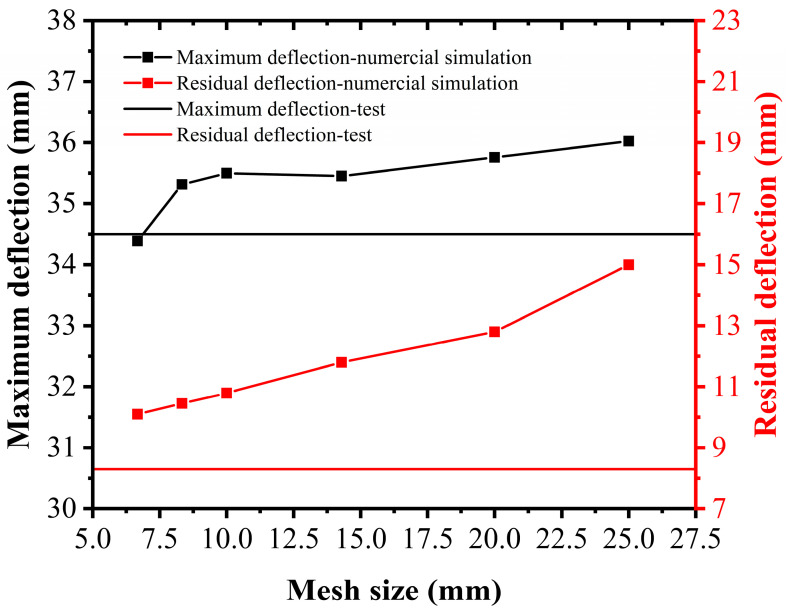
Effect of mesh size on the deflection of PUHPC-1 specimen.

To confirm the applicability and accuracy of the current mesh size, taking the PUHPC-1 specimen as an example, the mesh sizes of the UHPC and steel reinforcement were set as 6.67 mm, 8.33 mm, 10 mm, 14.29 mm, 20 mm, and 25 mm. Due to the assumption of full bonding behavior between UHPC and polyurea, the mesh thickness of polyurea was always 2 mm. Figure 12 shows the maximum and residual deflections of the PUHPC-1 specimen with different mesh sizes. The deviation between the test result and numerical simulation was small when the mesh size of the UHPC and steel reinforcement was in the range of 6.67 mm to 10 mm, and a mesh size of 10 mm ensured excellent simulation efficiency. Therefore, the mesh size of 10 mm was reasonable in the current FE model.

## 3. Discussion on the Strengthening Effectiveness of Polyurea

The developed benchmark FE model described above was further employed to conduct a comprehensive parametric analysis of the strengthening effects of polyurea on the blast resistance of UHPC slabs when the scaled distance, polyurea thickness, span-to-depth ratio of the slab, and longitudinal reinforcement ratio were varied. In the current study, the span-to-depth ratio was defined as the effective length of the UHPC slab divided by its thickness.

For the FE model in the parametric study, it is noted that the boundary conditions were the same as those in the field blast test, and at the same time, the blast loading was generated by the CONWEP model. The detonation point was fixed at a height of 0.63 m from the center of the slab; therefore, the TNT charge weight was adjusted to represent the different scaled distances of the burst. In addition, all the material properties (UHPC, polyurea, reinforcement, and supports) were kept the same as that in the benchmark FE model, and for all the cases, the rear face of the UHPC slab was strengthened by the polyurea.

### 3.1. Influence of the Scaled Distance

**Figure 13:**
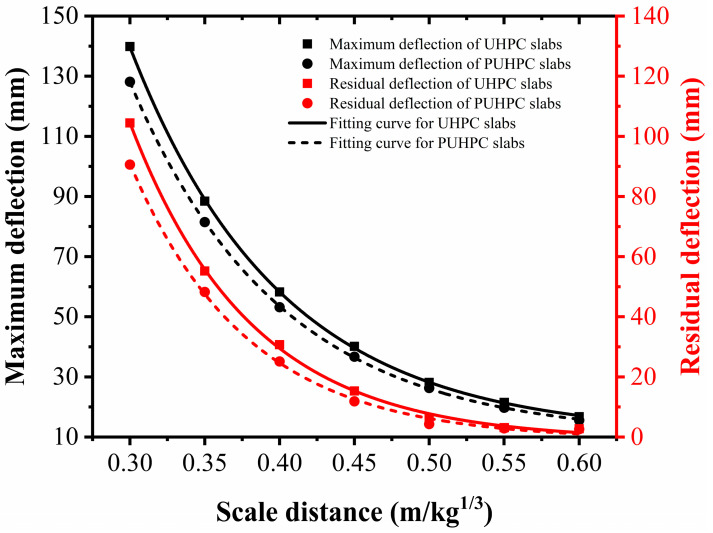
Influence of scaled distances on deflection for UHPC and PUHPC slabs.

In this section, the scaled distance was changed from 0.6 m/kg^1/3^ to 0.3 m/kg^1/3^, with an interval of 0.05 m/kg^1/3^. The span–depth ratio of the slab was 20, and the polyurea thickness was fixed at 4 mm in the FE simulation. Figure 13 reports the deflection of the slabs for varied scaled distances. From the figure, it is observed that the difference between the deflection curves for the UHPC and PUHPC slabs increased as the scaled distance decreased, implying that the reduction effects of polyurea on the maximum and residual deflections of the PUHPC slabs were more prominent as the scaled distance decreased. It is possible that the tensile hardening of the polyurea was completely triggered at a small-scale distance, thereby contributing to improving the anti-blast performance of the UHPC slabs. For the cases under scaled distances ranging from 0.6 m/kg^1/3^ to 0.5 m/kg^1/3^, the deflection of the slabs was observed to be at a low level, in which the maximum and residual deflections of the slab were found to be less than 30 mm and 10 mm, respectively. Therefore, the strengthening effectiveness of spraying polyurea was not prominent. However, even at a low level of deformation, the reduction ratio of the maximum deflection was larger than that of the residual deflection, indicating that spraying polyurea can serve to improve the initial bending stiffness of the slab. When the scaled distance varied from 0.4 m/kg^1/3^ to 0.3 m/kg^1/3^, the reduction effect of polyurea on the residual deflection was more significant than that on the maximum deflection. In particular, for a scaled distance of 0.4 m/kg^1/3^, the maximum and residual deflections of the UHPC slab were reduced by 5.08 mm and 5.57 mm, respectively. As the scaled distance further decreased to 0.3 m/kg^1/3^, the maximum and residual deflections of the UHPC slab were reduced by 11.72 mm and 13.86 mm, respectively. This phenomenon indicated that the polyurea can significantly improve the post-peak performance when the UHPC slab suffered a large deformation, that is, the strengthening effectiveness of spraying polyurea would be better with the increase in the deformation of the UHPC slab.

**Figure 14:**
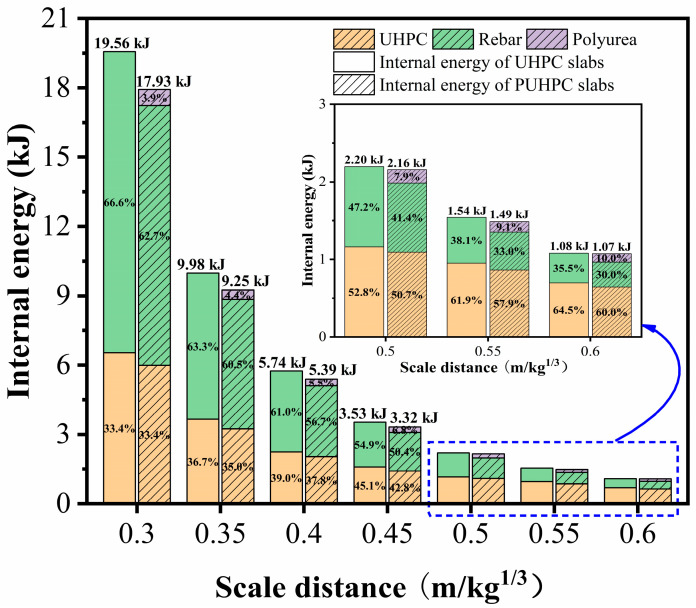
Influence of scaled distances on internal energy for UHPC and PUHPC slabs.

The absorption of the internal energy of UHPC and PUHPC slabs subjected to varied scaled distances is illustrated in Figure 14. Under the same scaled distance, the total internal energy absorption of the PUHPC slab was lower than that of the UHPC slab, and furthermore, the reduction in the absorbed internal energy for the PUHPC slab increased as the scaled distance decreased, indicating that spraying polyurea on the bottom of the UHPC slab can contribute significantly to distributing the blast energies. When the scaled distance was within the range of 0.5 m/kg^1/3^ to 0.6 m/kg^1/3^, the internal energy absorption for the UHPC material was higher than the total internal energy absorption of rebar and polyurea, implying that the UHPC material was the main component sustaining blast loading when the PUHPC slab exhibited an overall deformation at a small level. Therefore, under such circumstances, the strengthening effectiveness of polyurea was limited. However, for scaled distances smaller than 0.5 m/kg^1/3^, the absorbed internal energy for the UHPC material tended to be lower than the total absorbed internal energy of rebar and polyurea. It is possible that, under the condition of a small-scale distance, the slab may suffer a large bending deformation. Due to good adhesion between the polyurea layer and UHPC subbase, the tensile hardening of the polyurea can be completely developed, thereby leading to the improvement of the blast resistance of the UHPC slab. In addition, the absorbed internal energy of the polyurea increased as the scaled distance decreased, further demonstrating that the strengthening effect of spraying polyurea was more obvious when the scaled distance decreased.

### 3.2. Influence of the Polyurea Thickness

In this section, the polyurea thickness was changed from 4 mm to 16 mm, with an interval of 2 mm. Additionally, the span-to-depth ratio and the scaled distance were fixed at 20 and 0.3 m/kg^1/3^, respectively, and the longitudinal reinforcement ratio was consistent with that in the field blast test.

**Figure 15:**
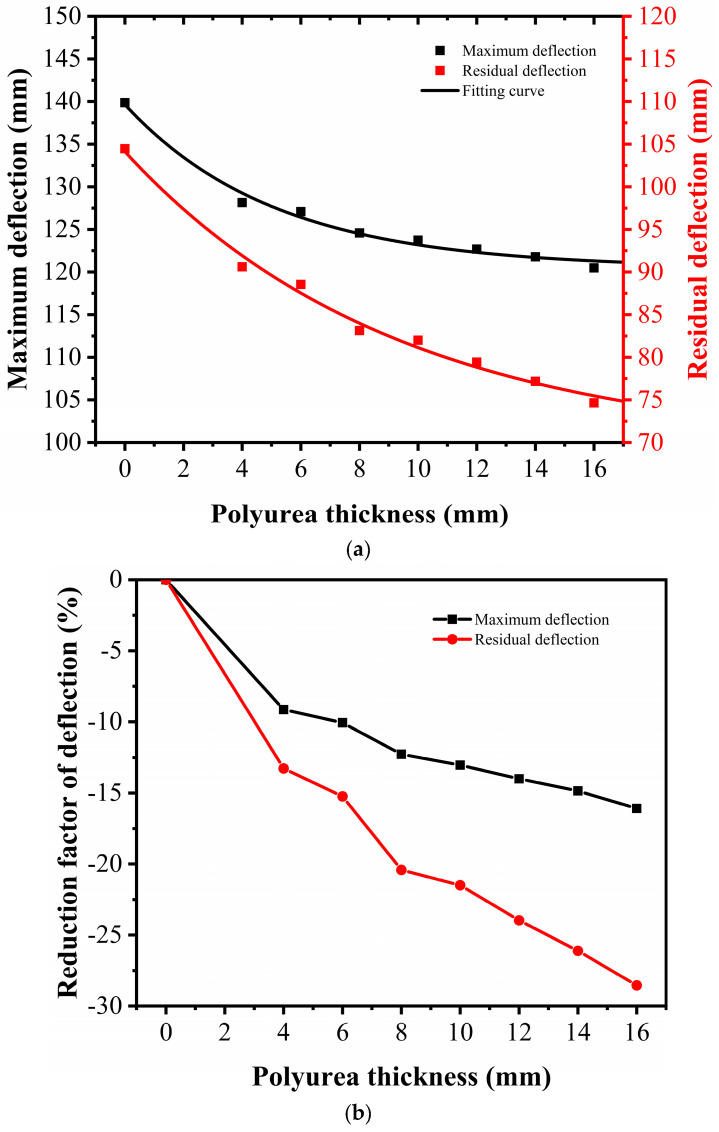
Influence of polyurea thicknesses on deflection for UHPC and PUHPC slabs: (**a**) Deflection (**b**) Reduction factor.

Figure 15a summarizes the maximum and residual deflections of the UHPC slab strengthened by polyurea with different thicknesses. In the figure, the slope of the curves can represent the effectiveness of the polyurea thickness on the reduction of the deflection of the UHPC slab. Figure 15a demonstrates that the slope of the maximum deflection curve tended to be stable for polyurea thicknesses exceeding 8 mm, indicating that the thickness of polyurea reached the threshold for strengthening. For the residual deflection, the curve exhibited a continuous decrease as the polyurea thickness increased, implying that the post-peak behavior of the UHPC slab was sensitive to the presence of polyurea. Figure 15b further illustrates the reduction factor of the deflection of the slab strengthened with different polyurea thicknesses, in which the reduction factor of deflection was defined as: (deflection of UHPC slab—deflection of PUHPC slab)/deflection of UHPC slab. According to the figure, the reduction factors of the maximum and residual deflections for the UHPC slab strengthened with 4 mm-thick polyurea were 9.15% and 13.26%, respectively. As the polyurea thickness reached 16 mm, the reduction factors for the maximum and residual deflections of the slab were 16.09% and 28.54%, respectively. It can be concluded that spraying polyurea had a better reduction effect on the residual deflection of the slab than on the maximum deflection.

**Figure 16:**
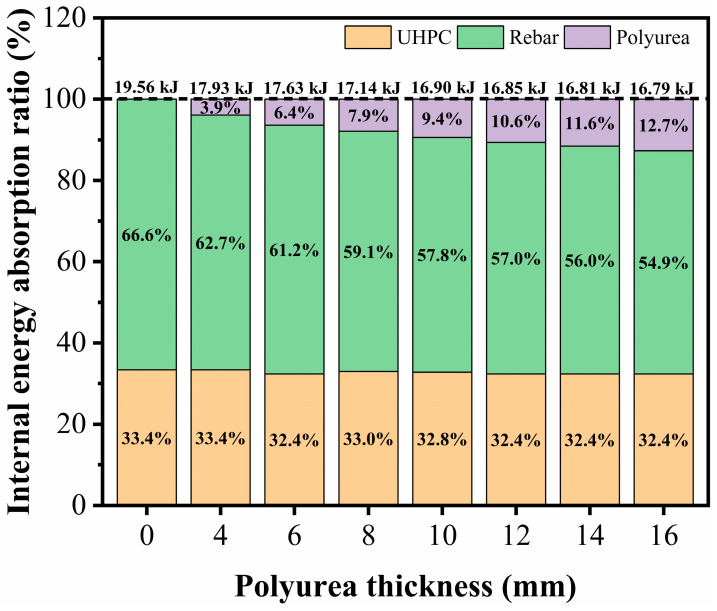
Influence of polyurea thicknesses on internal energy for UHPC and PUHPC slabs.

Figure 16 summarizes the internal energy absorption for the UHPC material, rebar, and polyurea under different polyurea thicknesses. The total absorbed internal energy of the UHPC material and rebar decreased with increasing polyurea thickness. However, the total internal energy absorption of the UHPC material and rebar for the slab with a polyurea thickness of 12 mm was close to that of 14 mm, demonstrating that the threshold of the polyurea thickness was 12 mm for the UHPC slab with a span-to-depth ratio of 20. In addition, as the polyurea thickness increased, the internal energy of the UHPC material and rebar tended to decrease, while the internal energy for the polyurea increased from 3.9% to 12.7%. This observation implied that spraying polyurea can effectively improve the flexural stiffness of the UHPC slab under dynamic loads and further reduce the damage of the specimen. Therefore, for the specimen with a span-to-depth ratio of 20, a reasonable polyurea thickness for blast mitigation of the UHPC slab was suggested to be in the range of 8 mm to 12 mm.

### 3.3. Influence of the Span-to-Depth Ratio of the UHPC Slab

In this section, the span-to-depth ratio for the UHPC slabs was selected to be 20, 24, 28, 32, and 36, while the scaled distance in the FE simulation was kept at 0.3 m/kg^1/3^. In the analysis, the change in the span-to-depth ratio for the UHPC slab was controlled by adjusting the effective length of the specimen, and three polyurea thicknesses (i.e., 4 mm, 8 mm, and 12 mm) were considered.

**Figure 17:**
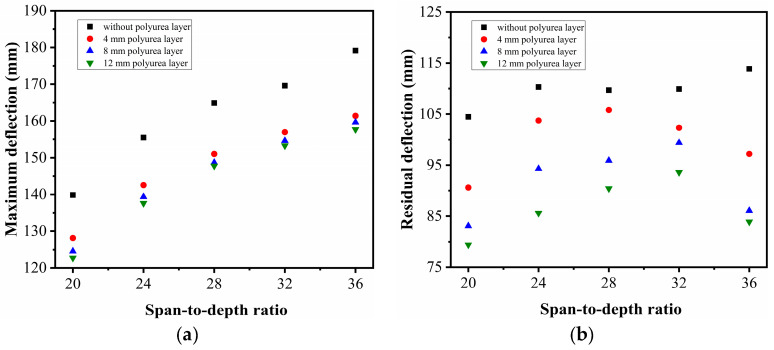
Influence of polyurea thicknesses on deflection for UHPC and PUHPC slabs with different span-to-depth ratios: (**a**) Maximum deflection (**b**) Residual deflection.

**Figure 18:**
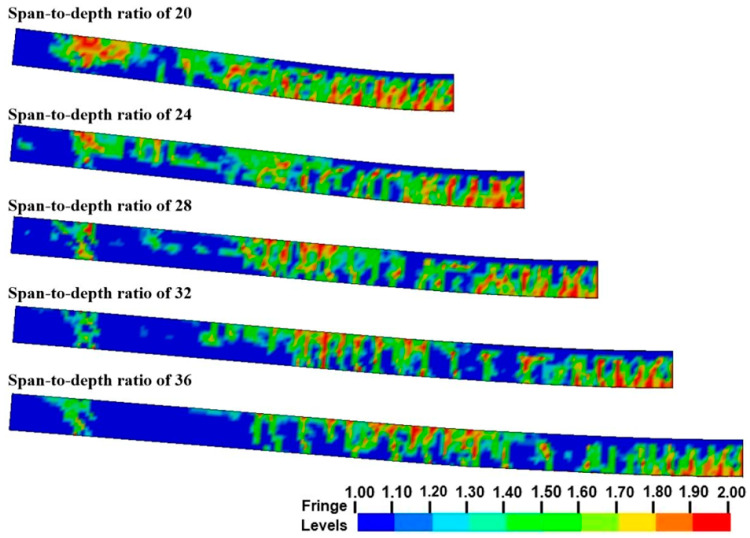
Damage pattern of UHPC slabs with different span-to-depth ratios.

Figure 17 shows the relationship between the deflection of the slab and the span-to-depth ratio for the slab strengthened by polyurea with different thicknesses. The figure illustrates that the maximum deflection of the slab exhibited an increasing tendency as the span-to-depth ratio increased. Additionally, it was shown that, for the UHPC slabs sprayed with a polyurea thickness of 4 mm to 8 mm, their maximum deflections were significantly reduced. However, when the polyurea thickness varied from 8 mm to 12 mm, the change in the maximum deflection of the slab was not sensitive to the polyurea thickness. At the same time, there was no obvious rule in the reduction of the residual deflection of the PUHPC slab. This may be because the damage pattern and failure mode were mainly affected by the span-to-depth ratio of the UHPC slab. Figure 18 further demonstrates the damage pattern of the UHPC slabs with different span-to-depth ratios. It is noted that, to clearly present the difference in the damaged mode between the slabs, only half of the FE model was given. From the figure, it was found that, for span-to-depth ratios ranging from 20 to 28, the UHPC slabs mainly exhibited mixed flexural-shear damage, in which shear failure tended to occur at the support. When the span-to-depth ratio of the slab increased from 28 to 36, the damage mode of the slab was shifted from shear failure into flexural failure, in which the shear damage at the support was significantly reduced. Therefore, it can be postulated that the strengthening effectiveness of polyurea was prominent for the reduction of the residual deflection when the UHPC slab mainly suffered flexural damage. Furthermore, by adopting the end rotation as an evaluation index (i.e., maximum deflection of the slab divided by the span of the slab), it was shown that under identical scaled distances, for span-to-depth ratios of 20, 24, 28, 32, and 36, the corresponding end rotations that occurred in the slab were 7.97°, 7.39°, 6.72°, 6.05°, and 5.69°, respectively. Therefore, it is obvious that the damage degree of the UHPC slab was reduced by spraying polyurea.

**Figure 19:**
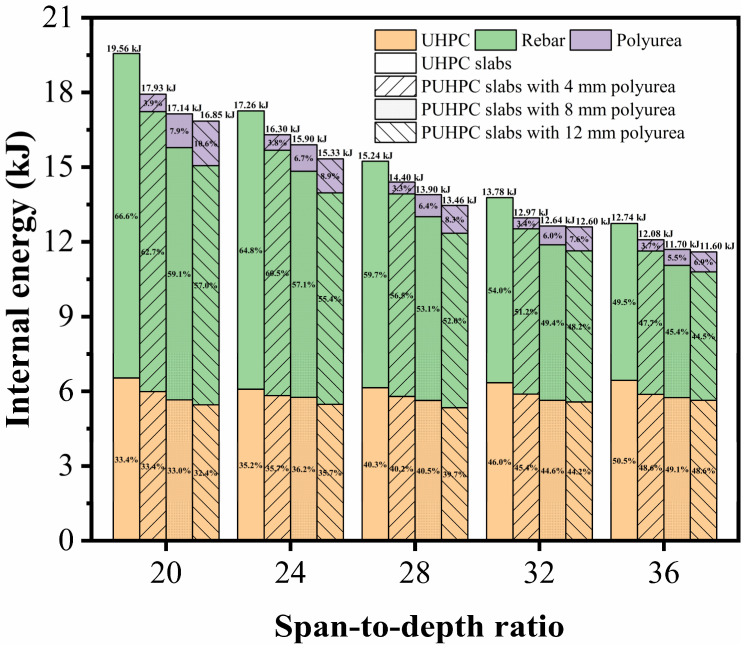
Influence of polyurea thicknesses on internal energy for UHPC and PUHPC slabs with different span-to-depth ratios.

Figure 19 quantifies the internal energy absorption of each component in the slab. The total absorbed internal energy of the slab gradually decreased when the span-to-depth ratio increased. It is possible that a larger span-to-depth ratio of the slab tended to make the overall response of the slab more intense, in which more cracks developed to release energy. For the UHPC and PUHPC slabs with identical span-to-depth ratios, it was found that the internal energy absorption of the polyurea increased as the polyurea thickness changed from 8 mm to 12 mm, implying that the strengthening effectiveness of the polyurea was more sensitive to the polyurea thickness. However, it was also found that the reduction in the total absorption internal energy of the slab decreased as the span-to-depth ratio increased, indicating that the strengthening effect of polyurea became limited. In addition, the internal energy absorption of the rebar decreased, demonstrating that spraying polyurea can improve the flexural stiffness of the slab. Hence, in terms of the results of the total internal energy absorption for the slab with a span-to-depth ratio varying from 20 to 36, it can be concluded that the optimum polyurea thickness was in the range of 8 to 12 mm.

### 3.4. Influence of the Longitudinal Reinforcement Ratio

In this section, three longitudinal reinforcement ratios for the UHPC and PUHPC slabs were considered, i.e., 0.63%, 1.12%, and 1.76%. In the FE simulation, the scaled distance and the span-to-depth ratio of the slab were set to 0.3 m/kg^1/3^ and 20, respectively, and the thickness of the polyurea layer was kept at 4 mm.

**Figure 20:**
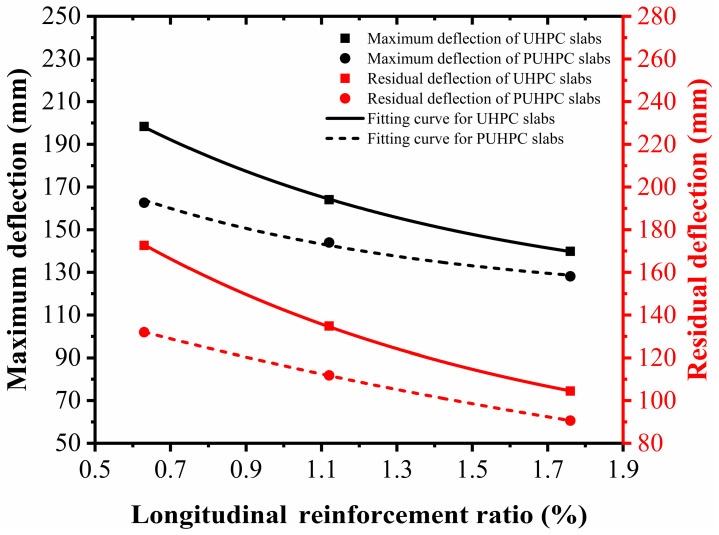
Influence of longitudinal reinforcement ratios on deflection for UHPC and PUHPC slabs.

Figure 20 shows the deflection of the slabs with different longitudinal reinforcement ratios. When the longitudinal reinforcement ratio of the slab increased from 0.63% to 1.76%, the maximum deflection of the slab strengthened with 4 mm-thick polyurea was reduced by 35.76 mm, 20.08 mm, and 11.72 mm, and the residual deflection was reduced by 40.63 mm, 22.98 mm, and 13.86 mm, respectively. This indicated that the polyurea had more prominent strengthening effectiveness on a UHPC slab with a lower longitudinal reinforcement ratio. Furthermore, in comparison with that of the maximum deflection, the reduction of the residual deflection was larger, demonstrating that, for a UHPC slab with a low reinforcement ratio, its post-peak behavior can be effectively improved by spraying polyurea on the rear face of the slab.

**Figure 21:**
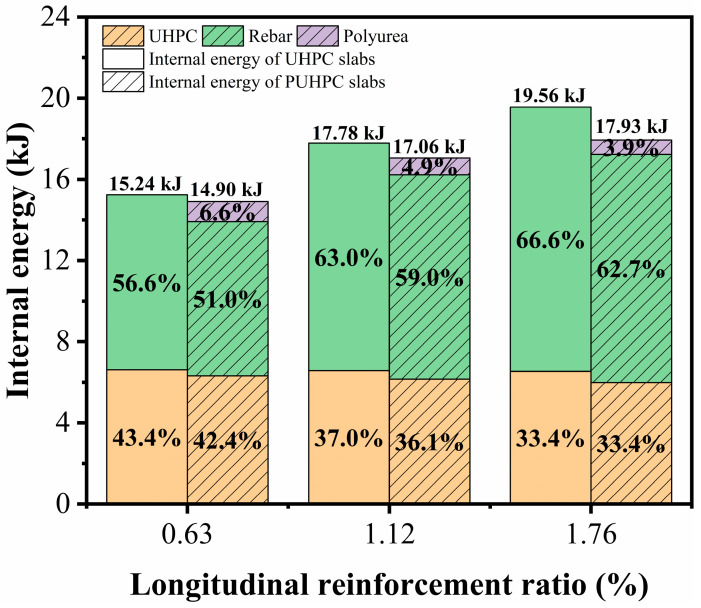
Influence of longitudinal reinforcement ratios on internal energy for UHPC and PUHPC slabs.

Figure 21 illustrates the absorbed internal energy of the UHPC and PUHPC slabs with different longitudinal reinforcement ratios. From the figure, it can be observed that the decrease in the longitudinal reinforcement ratio tended to reduce the absorbed internal energy of the rebar; therefore, the total internal energy of the slab decreased accordingly. Under the condition of the same longitudinal reinforcement ratio, the total internal energy absorption of the PUHPC slab was lower than that of the UHPC slab. This observation demonstrated that spraying polyurea can contribute to enhancing the blast resistance of the UHPC slab, thereby alleviating the damage degree of the UHPC slab. For the internal energy absorption of the rebar, it is shown that, with the reduction of the longitudinal reinforcement ratio, the absorbed internal energy of the rebar gradually decreased, whereas the internal energy absorption of the polyurea tended to increase. This phenomenon further demonstrated that the strengthening effectiveness of polyurea was suitable for a UHPC slab with a low longitudinal reinforcement ratio.

### 3.5. Prediction Formula

Based on the above parametric analysis, it can be found that factors including the scaled distance, polyurea thickness, span-to-depth ratio of the UHPC slab, and longitudinal reinforcement ratio in the UHPC slab would affect the deflection response of the UHPC and PUHPC slabs under blast loading. In this section, comprehensive FE simulations were further adopted to quantitatively investigate the combined effects of the four variables (the scaled distance, polyurea thickness, span-to-depth ratio, and longitudinal reinforcement ratio) on the anti-blast performance of polyurea-strengthened UHPC slabs. The four variables and their corresponding values used in this section are given in Table 9. In the following discussion, the end rotation of the slab was adopted as a damage index to compare the damage situation of the specimens with different configurations. The end rotation *θ* was defined as follows:

(3) $$\theta =\tan^{-1}(\frac{x_{\max}}{L_{\min}})$$

where *x*_max_ is the maximum deflection at the mid-span of the specimen and *L*_min_ is the nearest distance from the location of the maximum deflection to the support, which is equal to half of the effective span of the specimen in this study.

**Figure 22:**
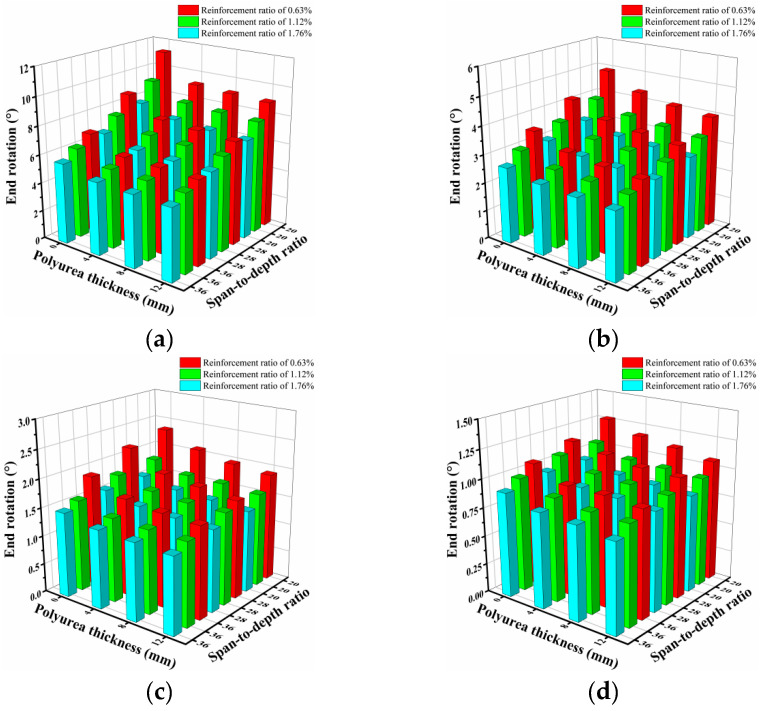
End rotation of UHPC and PUHPC slabs under different scaled distances: (**a**) *Z* = 0.3 m/kg^1/3^ (**b**) *Z* = 0.4 m/kg^1/3^ (**c**) *Z* = 0.5 m/kg^1/3^ (**d**) *Z* = 0.6 m/kg^1/3^.

Figure 22 shows the end rotations of the specimens with different configurations under blast loading and scaled distances ranging from 0.3 m/kg^1/3^ to 0.6 m/kg^1/3^. From the figure, it is obvious that, for the specimens under a small-scale distance (i.e., 0.3 m/kg^1/3^), spraying polyurea at the bottom of the UHPC slab can effectively reduce the end rotation, and the reduction tendency for the end rotation continuously develops as the polyurea thickness increases. For the same span-to-depth ratio, the end rotation of the slab progressively decreased as the longitudinal reinforcement ratio increased; however, the reduction trend for the end rotation decreased slowly with increasing polyurea thickness. For example, for the UHPC specimen with a span-to-depth ratio of 20 under a scaled distance of 0.3 m/kg^1/3^, when the longitudinal reinforcement ratio increased from 0.63% to 1.12% to 1.76%, the corresponding end rotation decreased from 11.22 to 9.32 to 7.96. However, for the specimen strengthened with a 12 mm-thick polyurea layer and with a span-to-depth ratio of 20 under a scaled distance of 0.3 m/kg^1/3^, when the longitudinal reinforcement ratio increased from 0.63% to 1.12% to 1.76%, the corresponding end rotation decreased from 8.97 to 7.95 to 7.00. In addition, as the span-to-depth ratio increased, the reduction trend of the end rotation became more stable. From Figure 22a–d, it can be seen that, when the specimens were subjected to blast loading with a larger scaled distance, the end rotation of the specimens with the same configurations decreased, in which the strengthening effectiveness of polyurea became less prominent.

The multivariate nonlinear algorithm was adopted to fit the results of the numerical simulation for predicting the end rotation *θ* of the slab under near-field explosion. The formula can be expressed as follows:

(4) $$\theta =\left[-4.711\times \left(\frac{s}{20}\right)-0.558\times \left(\frac{t}{4}\right)-4.047\times \left(\frac{r}{0.0176}\right)+20.979\right]\times \left(7.951e^{-8.556Z}+0.041\right)$$

where *s* represents the span-to-depth ratio (the range was defined from 20 to 36 in the present study), *t* represents the polyurea thickness (the range was defined from 0 mm to 12 mm in the present study), *r* represents the longitudinal reinforcement ratio (the range was defined from 0.0063 to 0.0176 in the present study), and *Z* is the scaled distance (the range was defined from 0.3 m/kg^1/3^ to 0.6 m/kg^1/3^ in the present study).

**Figure 23:**
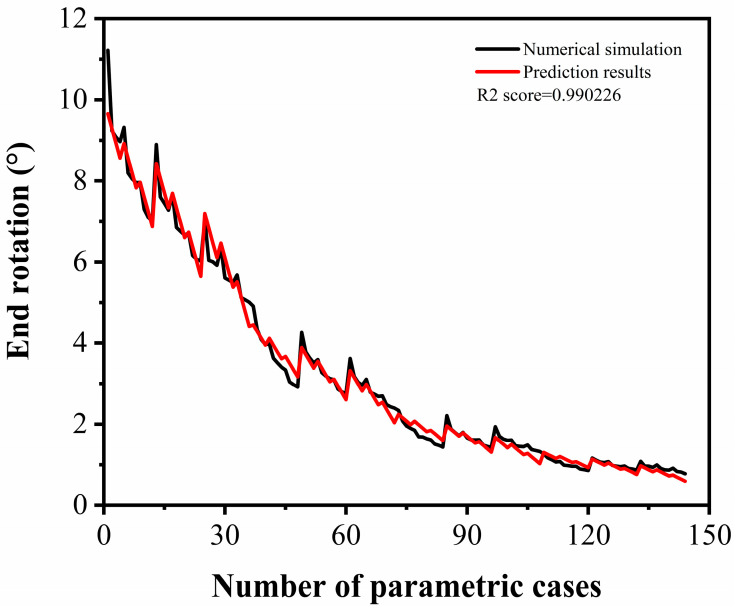
Comparison of end rotation between numerical simulation and prediction formula.

Figure 23 shows the comparison of the end rotation between the prediction formula and the numerical simulation. The prediction of the end rotation for the slab exhibited a good match with the numerical simulation. Hence, the fitted formula can be used to quickly predict the end rotation of UHPC slabs strengthened with different polyurea thicknesses under various near-field explosions.

## 4. Conclusions

In this paper, a benchmark FE model of UHPC and PUHPC slabs was established and validated with the actual recorded data from field blast tests. Parametric analysis was further carried out to investigate the strengthening effectiveness of polyurea for the blast mitigation of UHPC slabs by adopting the validated benchmark FE model. A prediction formula was then proposed to calculate the end rotation of the PUHPC slab under various blast loads. The conclusions were as follows:

(1) There was good agreement between the developed FE model and the field blast test results, in terms of the damage pattern and the deflection of the specimens. Therefore, the anti-blast response of the UHPC and PUHPC slabs under near-field explosion can be well-captured by the developed FE model and the parameters for the UHPC material model.
(2) The benchmark FE model was further adopted to investigate the key factors, including the scaled distance, polyurea thickness, span-to-depth ratio, and longitudinal reinforcement ratio, that influenced the anti-blast performance of the polyurea-strengthened UHPC slab. It was found that, with the decrease in the scaled distance, the strengthening effectiveness of polyurea would be more prominent, due to the large deformation of the specimen.
(3) The strengthening effectiveness of polyurea for the blast mitigation of the UHPC slab was also influenced by the span-to-depth ratio and longitudinal reinforcement ratio. When the deformation of the specimen was small, the strengthening effectiveness of polyurea for the blast mitigation of the UHPC slab was not prominent. As the deformation of the specimen increased (larger span-to-depth ratio or lower longitudinal reinforcement ratio), the strengthening effectiveness of polyurea increased to improve the anti-blast capacity of the UHPC slab, which was reflected in the reduction in the maximum deflection.
(4) Increasing the thickness of polyurea was a good choice for achieving excellent strengthening effectiveness for the blast mitigation of the UHPC slab. However, when the polyurea thickness exceeded a certain threshold value, further increasing the polyurea thickness would contribute less to improving the anti-blast performance of the UHPC slab. In terms of the deflection and the absorbed internal energy for the UHPC slabs strengthened with different polyurea thicknesses, the optimum thickness of polyurea for the blast mitigation of the UHPC slab was recommended to range from 8 mm to 12 mm.
(5) A fitting formula was established to predict the end rotation of a UHPC slab with or without sprayed polyurea via the multiple nonlinear regression method. The prediction formula also considered the key factors (i.e., the scaled distance, polyurea thickness, span-to-depth ratio, and longitudinal reinforcement ratio) that influenced the strengthening effectiveness of polyurea. Hence, the end rotation of the polyurea-strengthened UHPC slab can be quickly predicted for the purpose of damage assessment. However, more parameters (i.e., the boundary condition, width of the slab, and standoff distance of the explosive) should be taken into account to make the formula more suitable for the anti-blast design of UHPC components. It should also be noted that the established formula is not suitable for a polyurea-strengthened UHPC slab suffering combined flexural-shear failure.
(6) In the current study, the strengthening effectiveness of polyurea on a UHPC slab was numerically investigated under scaled distances ranging from 0.3 m/kg^1/3^ to 0.6 m/kg^1/3^. However, in actual circumstances, the structure can also be attacked under a smaller scaled distance or contact detonation. Therefore, it is necessary to further study the strengthening effectiveness of polyurea on a UHPC slab under a smaller scaled distance. In addition, a damage assessment criterion for a polyurea-strengthened UHPC slab should be further developed.

## Figures and Tables

**Table 1:** Mixing proportion of UHPC material in the current study (kg/m^3^).

Cement	Silica Fume	Superfine Mineral Admixture	Sand	Water	HRWR	Steel Fiber (by Volume)
700	140	110	1200	152	22.8	2%

HRWR is high-range water reducer.

**Table 2:** Mechanic properties of UHPC material.

Material	Compressive Strength (MPa)	Tensile Strength (MPa)	Elastic Modulus (GPa)	Poisson’s Ratio
UHPC	129	8.33	48.6	0.23

**Table 3:** Mechanic properties of steel reinforcement.

Number	Yield Strength (MPa)	Tensile Strength (MPa)	Elastic Modulus (GPa)	Tangent Modulus (MPa)	Poisson’s Ratio
Sample-1	495	608	185	925	0.3
Sample-2	457	586	181	808	0.3
Sample-3	454	560	185	860	0.3

**Table 4:** Mechanic properties of polyurea.

Material	Density (kg/m^3^)	Tensile Strength (MPa)	Elastic Modulus (MPa)	Poisson’s Ratio
Polyurea	1070	15	120	0.495

**Table 5:** Test program for the field blast test in present study.

Number	Specimen	Scaled Distance (m/kg^1/3^)	TNT Charge Weight (kg)	Distance to Face of the Slab (m)
1	UHPC-1	0.8	4	1.27
2	UHPC-2	0.5	4	0.79
3	PUHPC-1	0.6	4	0.95
4	PUHPC-2	0.5	4	0.79
5	PUHPC-3	0.4	4	0.63

**Table 6:** Parameters of strength surfaces of the UHPC material in present study.

**Parameter**	*a*_0_	*a*_1_	*a*_2_	*a*_0y_	*a*_1*y*_	*a*_2*y*_	*a*_1*f*_	*a*_2*f*_
**Value**	30.5686	0.3757	0.00115	24.5533	0.5007	0.00353	0.4125	0.000809

**Table 7:** Parameters of EOS for the UHPC in the current study.

Volumetric Strain	Pressure (MPa)	Bulk Modulus (MPa)
0	0	17,204
−0.0079	136.2	17,204
−0.0290	593.0	20,825
−0.0510	1071.0	29,793
−0.0628	1328.7	46,155
−0.0726	1540.8	76,016
−0.1260	2700.0	165,791
−0.2162	7925.6	172,043
−0.2880	11,089.7	172,043
−0.3597	13,757.7	172,043

**Table 8:** Comparison of deflection of the specimen between the experimental and FE results.

Specimen	Scaled Distance (m/kg^1/3^)	Maximum Mid-Span Deflection (mm)			Residual Mid-Span Deflection (mm)		
		Field Test Result (mm)	Numerical Results (mm)	Deviation from the Test (%)	Field Test Result (mm)	Numerical Results (mm)	Deviation from the Test (%)
UHPC-1	0.8	27.86	28.11	0.90	5.94	6.48	9.09
UHPC-2	0.5	-	48.41	-	28 *	22.60	−19.29
PUHPC-1	0.6	34.50	35.50	2.90	8.30	10.79	30.00
PUHPC-2	0.5	-	43.63	-	18.20 *	17.36	−4.62
PUHPC-3	0.4	-	55.31	-	30.30 *	26.96	−11.02

* Measured manually after blast testing.

**Table 9:** Parameters for comprehensive simulation.

Parameters	Span-to-Depth Ratio	Polyurea Thickness (mm)	Longitudinal Reinforcement Ratio	Scaled Distance (m/kg^1/3^)
Values	20, 28, 36	0, 4, 8, 12	0.63%, 1.12%, 1.76%	0.3, 0.4, 0.5, 0.6

## Data Availability

Not applicable.

## Appendix A

**Table A1:** Key parameters in KCC model (MAT_72R3) for UHPC material.

*** MAT_CONCRETE_DAMAGE_REL3**							
	RO	PR					
	0.00257	0.23					
FT	A0	A1	A2	B1	OMEGA	A1F	
8.33	30.5686	0.3757	0.00115	0.5	0.1	0.4125	
Sλ	NOUT	EDROP	RSIZE	UCF	LCRATE	LOCWID	NPTS
100	2.0	1.0	0.03937	145.0	Curve ID	25.4	13.0
λ01	λ02	λ03	λ04	λ05	λ06	λ07	λ08
0.0	4.4 × 10^−5^	7.0 × 10^−5^	7.48 × 10^−5^	9.5 × 10^−5^	1.2 × 10^−4^	2.1 × 10^−4^	3.4 × 10^−4^
λ09	λ10	λ11	λ12	λ13	B3	A0Y	A1Y
4.56 × 10^−4^	6.2 × 10^−4^	9.0 × 10^−4^	0.005	10.0	1.15	24.5533	0.5007
η01	η02	η03	η04	η05	η06	η07	η08
0.0	0.85	0.98	0.99	1.0	0.98	0.8	0.6
η09	η10	η11	η12	η13	B2	A2F	A2Y
0.5	0.4	0.3	0.07	0.0	−1.5	0.000809	0.00353

* The table format is the input format of LS-DYNA software.
